# Simplified Submucosal Tunneling Biopsy Using Clip-With-Line Traction and Closure for Gastric Subepithelial Lesion

**DOI:** 10.3390/diagnostics10090690

**Published:** 2020-09-12

**Authors:** Hideki Kobara, Nobuya Kobayashi, Noriko Nishiyama, Naoya Tada, Shintaro Fujihara, Tsutomu Masaki

**Affiliations:** Department of Gastroenterology and Neurology, Faculty of Medicine, Kagawa University, 1750-1 Ikenobe, Miki, Kita, Kagawa 761-0793, Japan; nobuyak@med.kagawa-u.ac.jp (N.K.); n-nori@med.kagawa-u.ac.jp (N.N.); n-tada@med.kagawa-u.ac.jp (N.T.); joshin@med.kagawa-u.ac.jp (S.F.); tmasaki@med.kagawa-u.ac.jp (T.M.)

**Keywords:** subepithelial lesion, EUS-guided fine needle aspiration, submucosal endoscopy, tissue sampling

## Abstract

Endoscopic ultrasound (EUS)-guided fine needle aspiration (FNA) has emerged as a standard and convenient method for the sampling of subepithelial lesions (SELs). Immunohistological analysis is required to definitively distinguish mesenchymal tumors; however, EUS-FNA provides insufficient material to achieve this, especially for small SELs < 2 cm. We therefore previously reported a novel submucosal tunneling biopsy (STB) technique that utilizes endoscopic submucosal dissection (ESD) for sampling SELs. However, unresolved advanced technical issues have hindered its widespread application. Currently, a counter-traction technique is used to facilitate ESD. We here describe a technically simplified STB technique using clip-with-line traction for gastric SELs.

**Figure 1:**
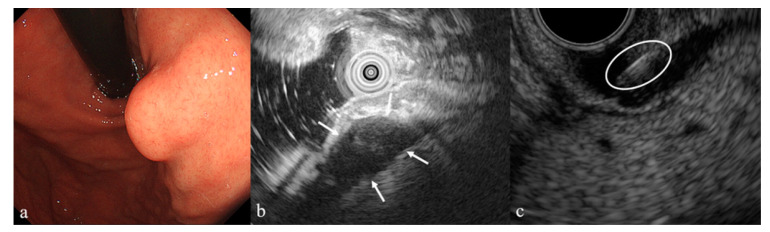
A 46-year-old man was referred to our department for evaluation of a gastric subepithelial lesion (SEL) measuring 15 × 9 mm, located in the cardia (**a**). Endoscopy revealed a rigid lesion with a negative cushion sign. Endoscopic ultrasound (EUS) showed a heterogeneous hypoechoic mass (white arrows) originating in the muscle layer (**b**), and computed tomography revealed an intraluminal growth pattern suspicious of gastrointestinal stromal tumor. Two needle (white circle) biopsies were obtained by endoscopic ultrasound-guided fine needle aspiration (EUS-FNA) (**c**); however, rapid on-site evaluation indicated that there were insufficient cells for cytologic examination. Our failure to obtain sufficient material, despite successful needle puncture, was probably attributable to the lesion’s small size and mobility. Thus, EUS-FNA has limited diagnostic accuracy for small SELs < 2 cm [1].

**Figure 2:**
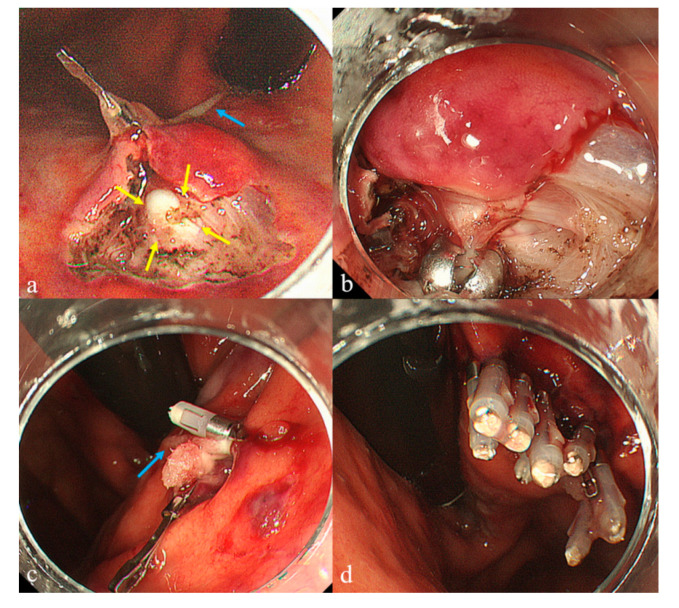
We have previously developed an original submucosal tunneling biopsy (STB) that involves submucosal endoscopy with a mucosal flap (SEMF) [2] for biopsying endoluminal SELs under direct vision [3]. The STB comprised five major steps: marking three dots, creating a 10-mm entry, creating a submucosal tunnel, tissue sampling, and entry closure. Its advantage over other biopsy methods is conferred by the use of a submucosal tunnel with SEMF, which enables direct visualization of the tumor, enabling acquisition of a core specimen of sufficient size for immunohistological analysis and avoiding delayed complications associated with entry closure. However, this technique also has technical difficulties related to submucosal tunnel creation and clip closure of the entry. Therefore, we performed the following modified STB after obtaining written informed consent. The patient was placed under deep sedation with intravenous midazolam (0.05 mg/kg). Magnifying endoscope (GIF-H260Z; Olympus, Tokyo, Japan) was used in order to well-visualize the tumor during the procedure. First, we used a retroflexed approach. Creation of the submucosal tunnel was facilitated by applying clip-with-line (blue arrow) traction using dental floss connected to the side hole of a hemoclip (Zeoclip; Zeon Medical Inc, Tokyo, Japan) [4,5,6], resulting in the visualization of a whitish tumor (yellow arrows) surrounded by the muscle layer (**a**). The diagnostic ability of this method depends on the identification of the tumor itself and the capsule. Our previous reports presented that endoscopically visualized feature (EVF), which each SEL itself expresses color, shape and solidity, could be classified in each SEL [7,8] Mesenchymal tumors, including gastrointestinal stromal tumor (GIST) and leiomyoma, represent whitish, round, and rigid characteristics. The proportions of a visible capsule were 33% (7/21) in mesenchymal tumors and 39% (5/13) in GIST, respectively [8]. Even in the presence of the capsule, we assume that the tumor can be distinguished by the EVFs, especially color, and the reliable target biopsy can be achieved under direct vision. After a vessel around the tumor was managed using hemostatic forceps (FD-410 LR; Olympus, Tokyo, Japan), several tissue samples were obtained from the identified tumor using biopsy forceps (Radial Jaw™ 4 Standard Capacity; Boston Scientific, Tokyo, Japan) (**b**). When grasping the biopsy forceps, slippage occurred due to the round and rigid tumor. Thus, we added one break for the tumor (approximately 2 mm in diameter) with the needle knife, leading to the acquisition of a secure sample under direct vision. Line-assisted closure [9] was then performed. The hemoclip (HX-610-090L; Olympus, Tokyo, Japan) holding the dental floss line (blue arrow) was anchored on the anal side of the entry via a forward approach. Pulling on this line approximated the defect of the entry (**c**), enabling its easy closure with additional hemoclips (**d**). The procedure time was 30 min and there were no intra- or post-procedure-associated complications. Immunohistological analysis revealed c-KIT negative, α-smooth muscle actin and desmin positive tissue, resulting in a diagnosis of gastric leiomyoma. The clip-with-line modification overcame previous technical issues regarding both traction and closure. Meanwhile, EUS-FNA is considered a safe and convenient method. Currently, novel FNA needles and forward EUS endoscopes are being developed to improve the diagnostic ability of EUS-FNA. Thus, our strategy proposes that EUS-FNA must be the first option for the sampling of SELs, followed by this method as the second alternative for unsuccessful FNA cases. This simplified STB (Supplementary Video S1) technique may be an acceptable means of sampling SELs.

## Supplementary Materials

The following are available online at https://www.mdpi.com/2075-4418/10/9/690/s1, Video S1: Simplified STB.
